# Association between psychiatric disorders and constipation: A population-based cross-sectional study

**DOI:** 10.1097/MD.0000000000051313

**Published:** 2026-10-30

**Authors:** Jie Kang, Fangyu Liu, Xu Yang, Nan Sui

**Affiliations:** a Third Clinical College, Liaoning University of Traditional Chinese Medicine, Shenyang, China; b Department of Rehabilitation Medicine, Northern Theater Command General Hospital, Shengyang, China; c Department of Anorectal Diseases, The Third Affiliated Hospital of Liaoning University of Traditional Chinese Medicine, Shengyang, China.

**Keywords:** brain-gut axis, constipation, cross-sectional study, NHANES, psychiatric disorders

## Abstract

Constipation is a common gastrointestinal disorder that can negatively affect patients’ quality of life. Previous studies have reported a higher prevalence of constipation among patients receiving long-term antipsychotic treatment; however, the association between Psychiatric disorders (PD) and constipation remains unclear. This study explored the association between PD and constipation. This cross-sectional observational study used data from the National Health and Nutrition Examination Survey (NHANES) 2009 to 2010. Psychiatric disorder status was determined based on whether a participant in the NHANES was prescribed antipsychotic medication. Constipation was defined based on the Bowel Health Questionnaire as either self-reporting being “always” constipated during the past 12 months or having both a Bristol Stool Form Scale type 1 or 2 stool and fewer than 3 bowel movements per week. Multivariable logistic regression was used to evaluate the association between PD and constipation after adjustment for potential confounding factors. Subgroup analyses were performed to assess this association across different population subgroups. : Our study included a total of 5092 participants aged 20 and above who met the analysis criteria. Among them, 343 participants met the criteria for constipation, corresponding to a survey-weighted prevalence of 5.5%. The survey-weighted prevalence of constipation was 9.6% among participants with PD and 4.9% among those without PD. After adjustment for potential confounding factors, PD were significantly associated with higher odds of constipation (OR = 1.767; 95% CI: 1.151–2.713; *P* = .024). Subgroup analyses showed statistically significant associations in several population subgroups; however, no statistically significant interactions were observed across the stratification variables. This cross-sectional study indicates a positive association between PD and constipation, providing evidence to inform clinical patient management.

## Graphical abstract

**Figure:**
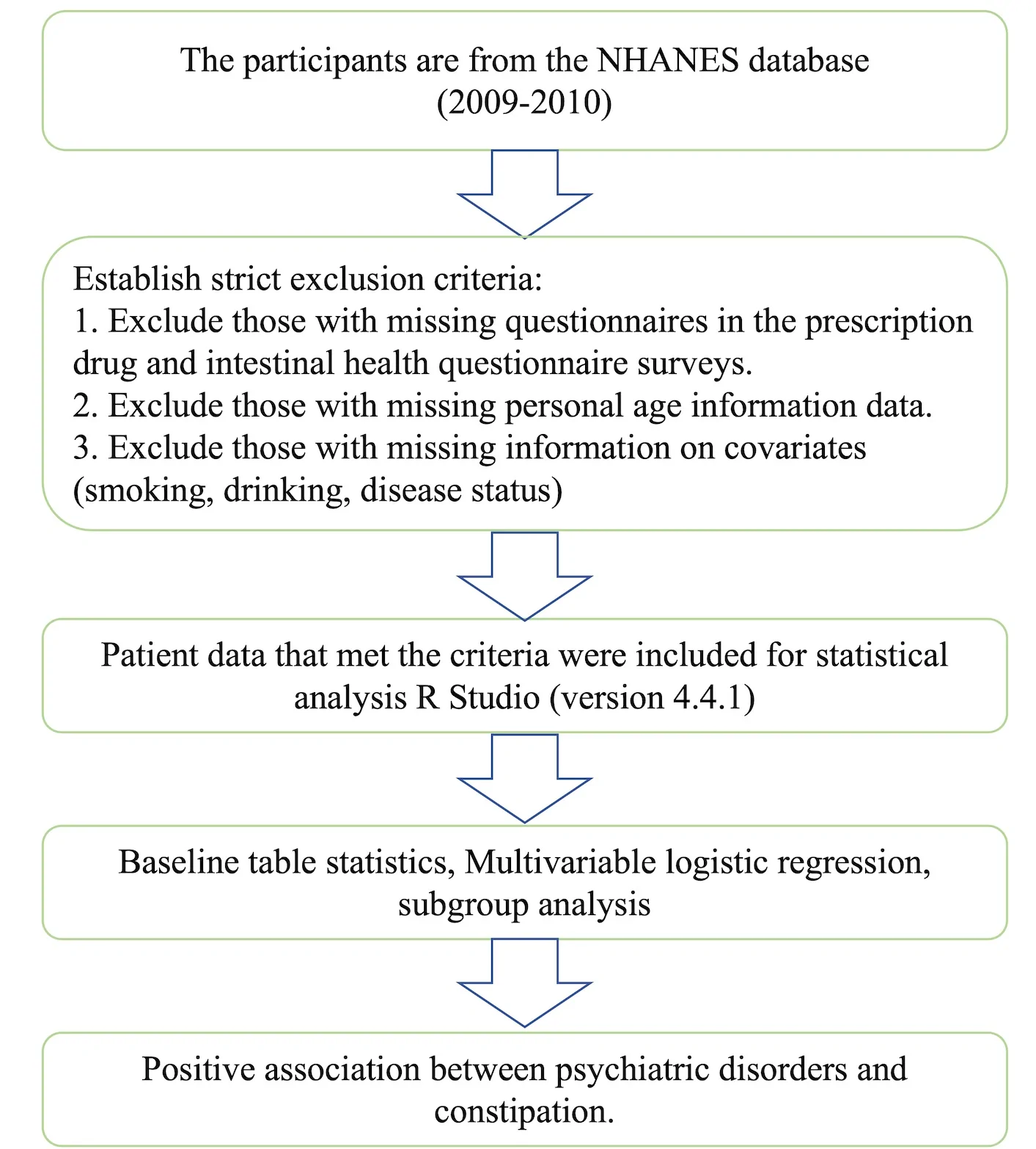

## 1. Introduction

Constipation (CN) is a common gastrointestinal disorder characterized by infrequent or difficult defecation, hard or dry stools, excessive straining, a sensation of incomplete evacuation, and anorectal blockage. Some patients may also require manual maneuvers to facilitate defecation, which can substantially impair quality of life.^[1]^ Epidemiological studies indicate that constipation affects approximately 15% of the global population, with the prevalence in women being nearly twice that in men.^[2,3]^ Constipation patients are often treated with oral fiber supplements, osmotic laxatives or probiotic preparations, among other treatments. However, previous studies have reported that fewer than half of patients achieve satisfactory symptom relief with conventional treatments.^[4]^ In the United States, there are approximately 8 million medical visits each year due to CN, with related medical expenses amounting to about 230 million US dollars, imposing a significant socio-economic burden.^[5,6]^ Therefore, identifying risk and protective factors associated with CN is important for its prevention and management. Current evidence suggests that constipation is associated with multiple pathophysiological mechanisms, including dysregulation of neurotransmitters, alterations in the gut microbiota, and impaired colorectal sensory function.^[7]^ In addition, depression, anxiety, chronic psychological stress, and the use of antipsychotic medications have been associated with constipation or worsening constipation-related symptoms.^[8]^

Psychiatric disorders (PD) refer to a group of conditions characterized by disturbances in cognition, perception, emotion, behavior, and other aspects of mental functioning that may result from the interaction of multiple biological, psychological, and environmental factors. PD represent a substantial global public health burden.^[9]^ Their clinical manifestations commonly include disturbances in cognition, thought processes, and emotional regulation, often accompanied by neurophysiological abnormalities and impaired social functioning.^[10]^ Among adults without PD, the prevalence of CN has been reported to range from approximately 2 to 27%, whereas a substantially higher prevalence, ranging from approximately 20 to 37%, has been reported among patients with PD.^[11,12]^ Mendelian randomization analysis has identified a positive correlation between schizophrenia, a mental disorder, and constipation.^[13]^ However, the relationship between PD and constipation has not yet been fully elucidated. Therefore, this study aims to explore the association between mental illness and constipation from multiple dimensions based on adult samples from the National Health and Nutrition Examination Survey (NHANES) in the United States, by adjusting for multiple potential confounding factors.

## 2. Materials and methods

### 2.1. Study participants

Data for this study were obtained from the NHANES (https://www.cdc.gov/nchs/nhanes/), a nationally representative survey conducted by the National Center for Health Statistics (NCHS). NHANES collects health-related information from the American population through standardized interviews, physical examinations, and laboratory tests. The NHANES has the following objectives: comprehensively assess the health conditions of adults and children across the United States; assess the national prevalence and related risk factors of common chronic diseases and infectious diseases in the United States; and to provide evidence for the development of public health and disease prevention strategies. The NHANES protocols were approved by the NCHS Research Ethics Review Board, and written informed consent was obtained from all participants at the time of the original NHANES survey.^[14]^ The present study was a secondary analysis of publicly available data from the NHANES database. Relevant publicly available data from the 2009 to 2010 NHANES cycle were used in this study (n = 20,905). Participants aged < 20 years were excluded because the Bowel Health Questionnaire (BHQ) was administered only to adults aged 20 years and older. Additional exclusion criteria were incomplete Prescription Medication Questionnaire data (n = 10,376), incomplete BHQ data (n = 5301), and missing data on relevant covariates (n = 136).^[15]^ Ultimately, a total of 5092 people participated in this study. The screening process of the research population is shown in Figure 1.

**Figure 1.**
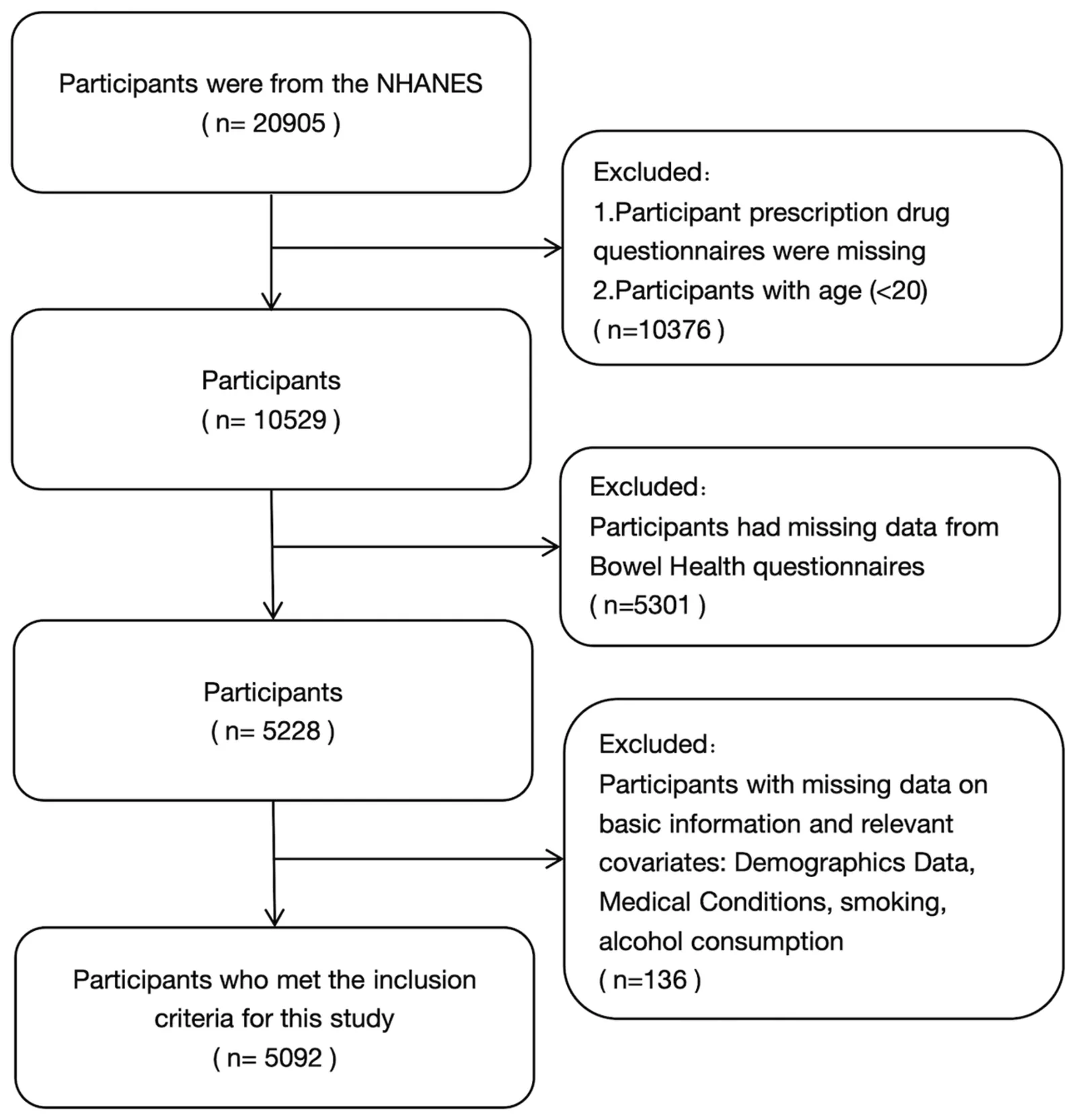
Flow chart of this study.

### 2.2. Evaluation of constipation

Data on bowel health were obtained from the BHQ in the NHANES database. The Mobile Examination Center (MEC) is a specially equipped mobile facility used by NHANES to conduct standardized health interviews, physical examinations, and laboratory assessments across different locations in the United States. The MEC interview component of the BHQ collected self-reported information on bowel habits and defecation from participants aged 20 years and older. In the present study, stool consistency and bowel movement frequency were used as indicators of CN. Stool consistency was assessed using the Bristol Stool Form Scale (BSFS), which was included in the NHANES BHQ. Participants were asked to select the stool type that most closely resembled their usual stool form. BSFS type 1 represents separate hard lumps, similar to nuts, whereas type 2 represents a sausage-shaped but lumpy stool; both types are commonly associated with CN.^[16]^ BSFS types 3 and 4 are generally considered to represent normal stool consistency, type 5 represents soft blobs with clear-cut edges, and types 6 and 7 represent loose or watery stools that are commonly associated with diarrhea. Participants were also asked to report their usual weekly bowel movement frequency. Based on previous studies, fewer than 3 bowel movements per week was considered indicative of constipation, whereas a frequency of 3 to 21 bowel movements per week was considered within the normal range.^[17,18]^ For the purposes of this study, constipation was defined based on responses collected as part of the NHANES BHQ. Participants were considered to have constipation if they met either of the following criteria: Those who answered “always” were considered to have constipation; If the BSFS classification was type 1 or 2 and the frequency of defecation was fewer than 3 times per week, the participant was considered to have constipation for the purposes of this study.

### 2.3. Evaluation of psychiatric disorders

The NHANES included a questionnaire on participants’ use of prescription medications during the previous 30 days, and the names of the reported medications were recorded and coded. The codes and names of prescription antipsychotic medications related to the treatment of PD that were used in the present study are listed in the Supplementary Materials, Supplemental Digital Content 1. Whether participants had PD was determined by whether they were taking prescription antipsychotic medications.

### 2.4. Covariates

Based on previous observational studies and the Mendelian randomization analysis conducted by our research group, a positive association between PD and CN had been identified.^[13,19]^ In the present study, whether participants had PD was considered the independent variable, and whether they had constipation was considered the outcome variable. To reduce the potential influence of confounding factors, the following covariates were included based on previous studies and available NHANES data: age, gender, race, marital status, drinking, smoking, diabetes, and coronary heart disease.^[18,20]^ The covariate of race was divided into 5 categories: Mexican American, Other Hispanic, Non-Hispanic White, Non-Hispanic Black, and Other Race. Marital status was divided into 3 categories: married, formerly married, and never married. Drinking was categorized into drinkers and nondrinkers. If a participant had consumed at least 12 alcoholic beverages in a year, they were considered a drinker; otherwise, they were considered a nondrinker. Smoking was categorized into smokers and nonsmokers. If a participant had smoked at least 100 cigarettes in their lifetime, they were considered a smoker; otherwise, they were considered a nonsmoker. Information on diabetes and coronary heart disease was obtained from NHANES and was classified according to whether participants reported having been diagnosed by a doctor or other health professional.

### 2.5. Statistical analysis

Considering the complex survey design of NHANES, the 2-year MEC examination weight (WTMEC2YR) was applied in the statistical analyses in accordance with the NHANES analytic guidelines.^[21]^ Continuous variables were presented as mean ± standard deviation. *t*-tests were used for comparisons between groups. Categorical variables were presented as percentages and were compared using chi-square tests.^[22]^ Multivariable logistic regression was used to evaluate the association between PD and CN, with PD as the independent variable and CN as the outcome variable. Odds ratios and 95% confidence intervals were calculated. Three regression models were constructed. Model 1 included PD as the independent variable without adjustment for any covariates. Model 2 was adjusted for age, gender, and race. Model 3 was fully adjusted for age, gender, race, marital status, drinking, smoking, diabetes, and coronary heart disease. Subgroup analyses stratified by age, gender, race, drinking, smoking, diabetes, and coronary heart disease were further performed to evaluate the association between PD and CN across different population subgroups. All statistical analyses were performed using R Studio (version 4.4.1). A *P* value < .05 was considered statistically significant.

## 3. Results

### 3.1. Baseline characteristics

A total of 5092 participants were included in this study, with a survey-weighted mean age of 46.9 ± 16.7 years. Males accounted for 49.4% of the sample; 8.4% were Mexican American, 11.0% were non-Hispanic Black, 69.4% were non-Hispanic White, 5.0% were Other Hispanic, and 6.2% were of Other Race. Among all participants, 343 met the criteria for constipation, corresponding to a survey-weighted prevalence of 5.5%. The survey-weighted prevalence of constipation was 9.6% among participants with PD and 4.9% among those without PD, and the difference was statistically significant (*P* < .001). All other covariates, with the exception of drinking, differed significantly between the PD and non-PD groups (*P* < .05) (Table 1).

**Table 1:** Basic characteristics of participants according to psychiatric disorder status.

Characteristics	Total	Psychiatric disorders Yes	Psychiatric disorders No	*P*-value
	N = 5092	N = 585	N = 4507	
Age (yr)	46.89 ± 16.71	52.01 ± 15.25	46.14 ± 16.78	<.001
Gender, n (%)				
Male	2520 (49.4%)	214 (35.2%)	2306 (51.4%)	<.001
Female	2572 (50.6%)	371 (64.8%)	2201 (48.6%)	
Race, n (%)				
Non-Hispanic White	2510 (69.4%)	412 (85.6%)	2098 (67.0%)	<.001
Non-Hispanic Black	897 (11.0%)	62 (5.7%)	835 (11.7%)	
Mexican American	919 (8.4%)	47 (3.1%)	872 (9.2%)	
Other Hispanic	521 (5.0%)	50 (3.3%)	471 (5.3%)	
Other Race	245 (6.2%)	14 (2.3%)	231 (6.8%)	
Marital status, n (%)				
Formerly_married	1133 (18.0%)	194 (27.9%)	939 (16.5%)	<.001
Married	3077 (64.3%)	318 (61.3%)	2759 (64.7%)	
Never married	882 (17.8%)	73 (10.8%)	809 (18.8%)	
Smoking				
Yes	2344 (44.3%)	322 (49.2%)	2022 (43.6%)	.012
No	2748 (55.7%)	263 (50.8%)	2485 (56.4%)	
Drinking alcohol				
Yes	3757 (78.1%)	428 (78.5%)	3329 (78.0%)	.853
No	1335 (21.9%)	157 (21.5%)	1178 (22.0%)	
Diabetes				
Yes	615 (8.7%)	106 (12.8%)	509 (8.0%)	.007
No	4477 (91.3%)	479 (87.2%)	3998 (92.0%)	
Coronary heart disease				
Yes	205 (3.2%)	36 (5.2%)	169 (2.9%)	.002
No	4887 (96.8%)	549 (94.8%)	4338 (97.1%)	
Constipation				
Yes	343 (5.5%)	65 (9.6%)	278 (4.9%)	<.001
No	4749(94.5%)	520 (90.4%)	4229 (95.1%)	

Continuous variables are presented as survey-weighted means ± standard deviations (SDs). Categorical variables are presented as unweighted counts and survey-weighted percentages. *P* values were calculated using survey-weighted *t*-tests for continuous variables and Rao–Scott adjusted chi-square tests for categorical variables.

### 3.2. The association between PD and CN

Multivariable logistic regression was performed to evaluate the association between PD and CN (Table 2). Without controlling for any confounding factors, PD was significantly associated with higher odds of CN in Model 1 (odds ratio [OR] = 2.041; 95% confidence interval [CI]: 1.585–2.629; *P* < .001). In Model 2, after adjustment for age, gender, and race, the association remained statistically significant (OR = 1.776; 95% CI: 1.332–2.367; *P* = .001). Model 3 included all covariates considered in this study, including age, gender, race, marital status, drinking, smoking, diabetes, and coronary heart disease. After full adjustment, PD remained significantly associated with higher odds of CN (OR = 1.767; 95% CI: 1.151–2.713; *P* = .024).

**Table 2:** The relationship between psychiatric disorders and constipation.

	Model 1	Model 2	Model 3
	OR (95% CI) P*	OR (95% CI) P*	OR (95% CI) P*
Exposure	Psychiatric disorders		
No	Reference	Reference	Reference
Yes	2.041 (1.585, 2.629) <.001	1.776 (1.332, 2.367) .001	1.767 (1.151, 2.713) .024

Model 1: unadjusted model; Model 2 was adjusted for age, gender, and race; Model 3 was simultaneously adjusted for marriage (formerly_married, married, never married), smoking (yes or no), drinking (yes or no), diabetes (yes or no), and coronary heart disease (yes or no) on the basis of Model 2.

OR = odds ratio, 95% CI = 95% confidence interval.

* *P* < .05 was considered statistically significant.

### 3.3. Subgroup analysis

To further evaluate the association between PD and CN across different population subgroups, stratified analyses were performed according to gender, race, marital status, drinking, smoking, diabetes, and coronary heart disease (Table 3). A significant association between PD and CN was observed among females (OR = 1.839; 95% CI: 1.219–2.775; *P* = .015). In the race-stratified analyses, significant associations were observed among non-Hispanic White participants (OR = 1.716; 95% CI: 1.194–2.467; *P* = .010) and Other Hispanic participants (OR = 3.797; 95% CI: 2.402–6.003; *P* < .001). Significant associations were also observed among married participants (OR = 1.899; 95% CI: 1.277–2.826; *P* = .009), smokers (OR = 2.000; 95% CI: 1.236–3.237; *P* = .016), drinkers (OR = 1.854; 95% CI: 1.130–3.042; *P* = .026), participants without diabetes (OR = 1.858; 95% CI: 1.257–2.745; *P* = .012), and participants without coronary heart disease (OR = 1.802; 95% CI: 1.208–2.687; *P* = .015). No statistically significant interactions were observed between PD and any of the stratification variables (all *P* for interaction > .05), indicating that the association between PD and CN did not differ significantly across these subgroups.

**Table 3:** The association results between covariates and psychiatric disorders and constipation.

Variable	OR	(95% CI)	*P*-value	*P* for interaction
Gender				
Female	1.839	1.219, 2.775	.015	.478
Male	1.401	0.658, 2.985	.283	
Race				
Mexican American	1.843	0.690, 4.922	.184	.058
Non-Hispanic Black	1.228	0.630, 2.395	.464	
Non-Hispanic White	1.716	1.194, 2.467	.010	
Other Hispanic	3.797	2.402, 6.003	<.001	
Other Race	0.155	0.001, 29.80	.404	
Marry				
Formerly_married	1.437	0.697, 2.964	.254	.757
Married	1.899	1.277, 2.826	.009	
Never married	1.935	0.495, 7.570	.268	
Smoking				
Yes	2.000	1.236, 3.237	.016	.201
No	1.573	0.907, 2.728	.084	
Drinking alcohol				
Yes	1.854	1.130, 3.042	.026	.632
No	1.401	0.611, 3.214	.323	
Diabetes				
Yes	1.075	0.200, 5.766	.911	.363
No	1.858	1.257, 2.745	.012	
Coronary heart disease				
Yes	1.026	0.075, 14.05	.980	.501
No	1.802	1.208, 2.687	.015	

OR = odds ratio, 95% CI = 95% confidence interval.

## 4. Discussion

This study, based on the NHANES, adopted a cross-sectional design to explore the association between PD and CN. According to the prespecified inclusion and exclusion criteria, a total of 5092 eligible participants were included for statistical analysis. Based on multivariable logistic regression adjusting for potential confounders, combined with subgroup analyses and tests of interaction, there was a statistically significant positive association between PD and CN. This finding supports the association between PD and intestinal dysfunction observed in previous clinical studies and provides additional evidence for considering mental and gastrointestinal health together in clinical management. Subgroup analyses showed that the association between PD and CN remained statistically significant among females, married participants, smokers, drinkers, non-Hispanic White participants, Other Hispanic participants, participants without diabetes, and participants without coronary heart disease. However, no statistically significant interactions were detected across the stratification variables. Therefore, the subgroup findings should not be interpreted as evidence that the strength of the association differed significantly according to gender, race, marital status, drinking, smoking, diabetes, or coronary heart disease. Existing literature suggests that hormonal fluctuations in women may influence gastrointestinal motility, while differences in dietary patterns and lifestyle behaviors across racial groups may affect intestinal health.^[23,24]^ However, these potential explanations were not directly evaluated in the present study and cannot be verified using our cross-sectional data. Previous clinical studies have reported a high prevalence of constipation among patients with PD,^[19,25]^ yet the biological mechanisms linking these 2 conditions remain incompletely understood.

Several biological pathways proposed in previous studies may provide plausible explanations for the observed association between PD and CN, although none of these mechanisms were directly evaluated in the present NHANES analysis. The brain-gut axis represents an important bidirectional communication pathway between the central nervous system and the gastrointestinal tract.^[26]^ At the neuroendocrine level, dysregulation of the hypothalamic-pituitary-adrenal axis has been implicated in PD and may influence gastrointestinal function through alterations in cortisol signaling and autonomic regulation.^[27]^ At the inflammatory level, previous studies have reported increased levels of proinflammatory mediators, including IL-6 and TNF-α, in some patients with PD.^[28,29]^ These inflammatory mediators may interact with MAPK-related signaling pathways, including ERK, apoptosis signal-regulating kinase 1, and p38/MAPK, which have been implicated in intestinal inflammation, epithelial barrier dysfunction, and neuroinflammatory responses.^[30–35]^ In addition, alterations in the gut microbiota and microbial metabolites may represent another pathway linking psychiatric and gastrointestinal disorders. Previous studies have reported changes in beneficial and pro-inflammatory bacterial populations in patients with PD,^[36]^ together with alterations in short-chain fatty acid production.^[37]^ Such changes may influence intestinal motility, mucus secretion, barrier function, neurotrophic signaling, and 5-HT metabolism.^[38,39]^ These findings from previous studies provide biologically plausible hypotheses for the association identified in the present study.

The present study has several strengths. First, the study population was derived from NHANES, which provides standardized and comprehensive health information from a large U.S. population sample. Second, multivariable regression models were used to adjust for several potential confounding factors, and subgroup and interaction analyses were performed to further evaluate the consistency of the association between PD and CN. These findings may have implications for the prevention and clinical management of constipation among individuals with PD. The findings suggest that bowel health may warrant greater clinical attention among individuals with PD, particularly when gastrointestinal symptoms are present. However, the present study does not establish that PD cause constipation or that specific preventive or therapeutic interventions would improve clinical outcomes. Future longitudinal and interventional studies are needed to determine whether integrated assessment and management of mental and gastrointestinal health provide measurable clinical benefits.

Our research provides a theoretical basis for understanding the association between PD and CN; however, several limitations should be acknowledged. Firstly, due to the inherent limitations of cross-sectional studies, the causal relationship between PD and CN remains unclear. Therefore, the potential causal relationship between the 2 still requires further exploration through longitudinal studies.^[40,41]^ Secondly, the data selected for this study are mostly from questionnaire surveys and belong to the category of recall data. Although most large-scale population studies rely on this, some people still have potential recall biases. Such recall bias may attenuate the observed associations and affect the accuracy of the study findings. Future studies should consider more comprehensive assessment methods, such as prospective daily recording of defecation-related information, to reduce recall bias.^[42,43]^ Thirdly, the dataset selected for our research is mainly targeted at the American population and has certain population specificity. Therefore, the generalizability of these findings to populations in other countries requires further investigation. Finally, this study used NHANES 2009 to 2010 data because this cycle provided the bowel health variables required for our predefined constipation assessment. The age of the dataset is an important limitation, as demographic characteristics, lifestyle patterns, psychiatric treatment, and clinical practice may have changed over time. Therefore, the prevalence estimates observed in this study may not fully reflect the contemporary U.S. population. Nevertheless, the findings provide population-based evidence of an association between PD and CN. Future studies using more recent datasets with comparable bowel health measurements are needed to determine whether this association persists in current populations.^[44]^

Despite these limitations, the present study provides population-based evidence supporting an association between PD and CN. Future longitudinal and prospective studies incorporating more comprehensive assessments are warranted to clarify the temporal and potential causal relationships between these conditions and to further investigate the underlying biological mechanisms.

## 5. Conclusion

In this study, we observed a positive association between PD and CN among adults in the United States. This finding suggests that bowel health may warrant greater attention in the clinical management of patients with PD. Further longitudinal and clinical studies are needed to confirm this association and to clarify the underlying mechanisms.

## Acknowledgments

We sincerely thank the National Health and Nutrition Examination Survey (NHANES) for providing the publicly available data used in this study. We also thank the reviewers and editors for their constructive comments and suggestions, which helped improve the quality of the manuscript.

## Author contributions

**Conceptualization:** Jie Kang, Nan Sui.

**Data curation:** Jie Kang, Nan Sui.

**Investigation:** Jie Kang, Fangyu Liu, Nan Sui.

**Methodology:** Jie Kang, Fangyu Liu, Xu Yang, Nan Sui.

**Validation:** Nan Sui.

**Visualization:** Jie Kang, Xu Yang, Nan Sui.

**Writing – original draft:** Jie Kang, Fangyu Liu, Xu Yang.

**Writing – review & editing:** Jie Kang, Xu Yang, Nan Sui.


